# Reduction of hyperglycemia in STZ-induced diabetic mice by prophylactic treatment with heat-killed *Mycobacterium aurum*: possible effects on glucose utilization, mitochondrial uncoupling, and oxidative stress in liver and skeletal muscle

**DOI:** 10.3389/fendo.2024.1427058

**Published:** 2024-09-06

**Authors:** Farid Abdallah, Samer Bazzi, Charles Akle, Georges M. Bahr, Karim S. Echtay

**Affiliations:** ^1^ Department of Biomedical Sciences, Faculty of Medicine and Medical Sciences, University of Balamand, Al-Koura, Lebanon; ^2^ Immune Boost Clinic Limited, Saint Michael, Barbados

**Keywords:** diabetes, *Mycobacterium aurum*, uncoupling protein 2 (UCP2), uncoupling protein 3 (UCP3), GLUT2, GLUT4, oxidative stress, LDH

## Abstract

**Background:**

In addition to conventional treatment and modifications in physical activity and diet, alternative strategies have been investigated to manage, prevent, or delay diabetes in humans. In this regard, one strategy has relied on the immunomodulatory properties of mycobacteria, whereby Bacillus Calmette–Guerin, an attenuated live strain of *Mycobacterium bovis*, has been shown to improve glycemic control in patients with diabetes and to alleviate hyperglycemia in selected murine models of diabetes. A novel heat-killed (HK) whole-cell preparation of *Mycobacterium aurum* (*M. aurum*) is currently under development as a potential food supplement; nevertheless, its potential bioactivity remains largely unknown. Thus, the present study investigated the potential prophylactic anti-diabetic effects of HK *M. aurum* in streptozotocin (STZ)–induced diabetic mice.

**Methods:**

Mice were divided into three groups: the STZ-induced diabetic group was injected with a single intraperitoneal high dose of STZ, the HK *M. aurum*–treated diabetic group was prophylactically treated with three doses of HK *M. aurum* 6 weeks before STZ injection, and the control non-diabetic group was given three intradermal injections of borate-buffered saline and an intraperitoneal injection of citrate buffer. Liver lactate dehydrogenase (LDH), uncoupling protein 2 (UCP2), and glucose transporter 2 (GLUT2) and skeletal muscle LDH, UCP3, and GLUT4 protein expression levels in different mouse groups were determined by Western blot.

**Results:**

Our results indicated that HK *M. aurum* did not cause any significant changes in glycemic levels of normal non-diabetic mice. Prophylactic administration of three doses of HK *M. aurum* to diabetic mice resulted in a significant reduction in their blood glucose levels when compared to those in control diabetic mice. Prophylactic treatment of diabetic mice with HK *M. aurum* significantly restored their disturbed protein expression levels of liver UCP2 and LDH as well as of skeletal muscle UCP3. On the other hand, prophylactic treatment of diabetic mice with HK *M. aurum* had no significant effect on their liver GLUT2 and skeletal muscle GLUT4 and LDH protein expression levels.

**Conclusions:**

Our findings provide the first evidence that HK *M. aurum* possesses a hyperglycemia-lowering capacity and might support its future use as a food supplement for the amelioration of diabetes.

## Introduction

According to the International Diabetes Federation, diabetes has become a disease of pandemic proportions whereby it is projected to reach more than 1.31 billion people worldwide by 2050 (1). Given the global burden of diabetes, several studies have been looking for novel strategies to alleviate or prevent the symptoms and complications of diabetes such as the use of herbal products or probiotics (2, 3). Type 2 diabetes (T2D) can be largely prevented through diet and lifestyle changes; however, prevention of type 1 diabetes (T1D) remains challenging. Bearing in mind that T1D is an autoimmune disease during which the immune system attacks the pancreatic β-cells and insulin auto-antigens, researchers have attempted to identify an immunomodulatory agent for the prevention or treatment of this disease.

Mycobacterial species are known to hold a powerful immunomodulatory capability, and this is mainly attributed to their complex cell wall composition (4). In this context, exploiting such immunomodulatory properties might represent one innovative approach to reverse, delay, or prevent the onset of diabetes. Bacille Calmette–Guérin (BCG), a live attenuated strain of *Mycobacterium bovis* (*M. bovis*), has been administered to humans for more than 100 years as a vaccine against tuberculosis and later as a therapeutic agent for non-invasive bladder cancer (5). The potential use of BCG to prevent or manage diabetes has been demonstrated in several studies that were conducted in both humans and mice, whereby there were several variations among those studies in terms of BCG’s used strains, doses, as well as the route and timing of its administration (6–10). Recent epidemiological data strongly support a possible role for BCG in the prevention of T1D, but not T2D (11). Moreover, BCG has been previously shown to lower hyperglycemia in patients with T1D as well as in single high-dose streptozotocin (STZ)–induced diabetic mice (12). BCG exerts its hyperglycemia lowering effects in humans by inducing the expansion of regulatory T cells (Tregs) and reducing the numbers of cytotoxic T cells (Tcs). Interestingly, BCG also acts on T lymphocytes by promoting glycolysis, which leads to the consumption of high amounts of glucose, thus lowering hyperglycemia (12, 13).

Considering the uncommon adverse effects and rare complications (i.e., local abscess formation, regional lymphadenitis, and disseminated BCG disease) experienced with multiple BCG administration (5) and the current sporadic shortage in BCG production (14), an alternate immunomodulatory-based approach that relies on the use of an environmental mycobacterium may prove to be efficient in preventing or treating diabetes. *Mycobacterium vaccae* (*M. vaccae*) is an environmental, rapidly growing mycobacterium that has been used in the form of a heat-killed (HK) whole-cell preparation as an immunotherapeutic/immunomodulatory agent in various disorders and disease settings (15–17). Similar to BCG, HK *M. vaccae* has been revealed to induce the expansion of Tregs (18) and to immunomodulate innate immune cells (19). In a study employing whole-genome sequencing, the environmental and rapidly growing *Mycobacterium aurum* (*M. aurum*; also known as *Mycolicibacterium aurum* Aogashima) was positioned in close proximity to *M. vaccae* (20). Little is known about the immunomodulatory properties of *M. aurum*; however, a HK whole-cell preparation of this mycobacterial strain is currently undergoing development as a potential psychobiotic (21). HK *M. aurum* was previously reported to exhibit no toxicity or pathogenicity in rats following its oral administration, thus justifying its potential use as a food ingredient (22). Given its phylogenetic proximity to *M. vaccae*, *M. aurum* may also hold a yet undiscovered similar immunomodulatory potential that may be beneficial in the prevention or management of diseases, such as diabetes.

The present study aimed to evaluate the efficacy of HK *M. aurum* in preventing the development of diabetes in STZ-induced diabetic BALB/c mice. Therefore, we examined the effect of HK *M. aurum* on blood and urine glucose levels in diabetic mice. Moreover, we assessed in diabetic mice the effect of HK *M. aurum* on the expression of key proteins involved in glucose transport and metabolism in the liver and skeletal muscle which are insulin-sensitive tissues significantly influenced by insulin insufficiency and hyperglycemia (23).

## Materials and methods

### Antibodies

The following antibodies were used: goat anti-mouse uncoupling protein 2 (UCP2), rabbit anti-mouse UCP3 antibodies (Santa Cruz Biotechnology), rabbit anti-mouse lactate dehydrogenase (LDH) A subunit antibody (Abcam), rabbit anti-mouse glucose transporter 2 (GLUT2) antibody (EMD Millipore), mouse anti-mouse GLUT4 antibody (Cell Signaling), goat anti-mouse actin antibody, horseradish peroxidase (HRP)–conjugated donkey anti-goat antibody (Santa Cruz Biotechnology), HRP-conjugated donkey anti-rabbit antibody (Abcam), and HRP-conjugated goat anti-mouse antibody (Invitrogen).

### HK mycobacteria

Briefly, sterile HK *M. aurum* whole-cell preparations (stock: 200 mg/mL in water) were manufactured by autoclaving for 15 min at 121°C (kindly provided by Immune Boost Clinic Limited). Sub-dilutions were prepared in borate-buffered saline (BBS) to make solutions of 1 mg/mL and 10 mg/mL that were stored at 4°C for later administration to mice.

### STZ preparation

STZ (Sigma-Aldrich) was freshly made by dissolving in ice-cold citrate buffer (CB; pH 4.5), whereby a preparation of STZ (150 mg/kg), was prepared and administered to mice, within the first 30 min of STZ preparation (12).

### Animals

Adult male BALB/c mice (4–6 weeks in age) ranging in weight from 18 g to 25 g were used in all experiments. Mice were housed at the University of Balamand animal facility that is set with a 12-h dark/light cycle and fed regular chow *ad libitum*. The animal experimental protocols were approved by the research committee at the Faculty of Medicine and Medical Sciences, University of Balamand. The experiments with animals were performed in accordance with the guidelines for ethical conduct in the care and use of animals set by the institution. Experimental mouse groups were randomly assigned into nine groups that were matched by weight (20.8 ± 0.98 g) and age.

### Experimental design

Mice were randomly divided into nine matched groups. The control non-diabetic “BBS” group received three doses of 100 µL of BBS given 2 weeks apart over 6 weeks. The control non-diabetic “BBS + CB” group received three doses of 100 µL of BBS given 2 weeks apart over 6 weeks and a single dose of 100 µL of CB given on the sixth week after the first BBS dose. The “Ma (0.1)” group received three doses of 100 µL of 0.1 mg of HK *M. aurum* given 2 weeks apart over 6 weeks. The “Ma (0.1) + CB” group received three doses of 100 µL of 0.1 mg of HK *M. aurum* given 2 weeks apart over 6 weeks and a single dose of 100 µL of CB given on the sixth week after the first *M. aurum* dose. The “Ma (1)” group received three doses of 100 µL of 1 mg of HK *M. aurum* given 2 weeks apart over 6 weeks. The “Ma + CB” group received three doses of 100 µL of 1 mg of HK *M. aurum* given 2 weeks apart over 6 weeks and a single dose of 100 µL of CB given on the sixth week after the first HK *M. aurum* dose. The diabetic “BBS + STZ” group received three doses of 100 µL of BBS given 2 weeks apart over 6 weeks and a single dose of STZ (150 mg/kg body weight in 100 µL of CB per dose) given on the sixth week after the first BBS dose. The “Ma (0.1) + STZ” group received three doses of 100 µL of 0.1 mg of HK *M. aurum* given 2 weeks apart over 6 weeks and a single STZ dose (150 mg/kg body weight, in 100 µL of CB) given on the sixth week after the first HK *M. aurum* dose. The “Ma + STZ” group received three doses of 100 µL of 1 mg of HK *M. aurum* given 2 weeks apart over 6 weeks and a single STZ dose (150 mg/kg body weight, in 100 µL of CB) given on the sixth week after the first HK *M. aurum* dose. Mice pre-treated with BBS or HK *M. aurum* received intradermal (at the base of the tale) injections of either treatment. BBS was given as a vehicle control for HK *M. aurum* to the aforementioned assigned mouse groups. A single high-dose injection of STZ (150 mg/kg) was administered intraperitoneally to STZ-treated mice so as to induce hyperglycemia (Kuhtreiber et al., 2018). CB was given intraperitoneally as a vehicle control for STZ to mice in non-diabetic groups. Mouse groups injected with STZ received 10% sucrose water for the first 48 h after STZ injection. Experimental design and procedures are summarized in **Supplementary Figure 1**.

### Measurement of body weight, urine, and blood glucose levels

Mice body weights as well as their blood glucose levels were monitored every 2 weeks for a period of 6 weeks before STZ injection and on a weekly basis for a period of 6 weeks after STZ injection. Blood was collected from the tail vein of mice for measurement of blood glucose levels using the human Accu-Chek Guide glucometer (Roche Diagnostics). Mice were allowed to fast for 4 h prior to blood glucose measurements. Urine glucose levels in mice were measured before STZ injection at week 0 and on weeks 2 and 4 after STZ injection using urine colorimetric strips (Acon Laboratories).

### Western blot analysis

Total protein (50 µg) isolated from the liver and skeletal muscle 6 weeks after STZ injection was electrophoresed through 12% SDS–polyacrylamide gel and then electro-blotted to polyvinylidene difluoride (PVDF) membranes (GE Healthcare). The membranes were blocked with 5% bovine serum albumin (BSA) in 1% tris-buffered saline (TBS) containing 1% Tween 20 (1% TBS-T) and probed overnight with the primary antibody of choice (anti-UCP2, anti-UCP3, anti-GLUT2, anti-GLUT4, anti-LDH, or anti-actin antibody). Membranes were then washed five times for 10 min with 1% TBS-T, before incubation with the respective secondary antibody for an hour and a half under constant shaking. Membranes are then washed again (five times for 5 min). Finally, the membranes were incubated with Clarity Western enhanced chemiluminescence substrate (Bio-Rad) for 5 min at room temperature and visualized using the Chemidoc system (Bio-Rad). The bands were quantified using the Image lab software (version 5.2.1; Bio-Rad).

### Statistical analysis

Statistical analysis was performed using GraphPad Prism software (version 6; GraphPad Software). Data were presented as mean values ± standard error of the mean (SEM). Unpaired t-test with Welch correction was used to compare the means of two different experimental groups. One-way or two-way ANOVA test followed by the Tukey’s multiple comparison *post-hoc* test was used to compare the means between three different experimental groups. Multiple unpaired t-tests were used to compare the means between two different experimental groups at different time points. Each P-value is adjusted to account for multiple comparisons. Differences between groups were considered to be statistically significant at *p* < 0.05. The schematic diagram was created using Biorender website.

## Results

### HK *M. aurum* prophylactic treatment does not induce hypoglycemia or weight change in non-diabetic mice but reduces hyperglycemia and glucosuria in STZ-induced diabetic mice

**Figure 1:**
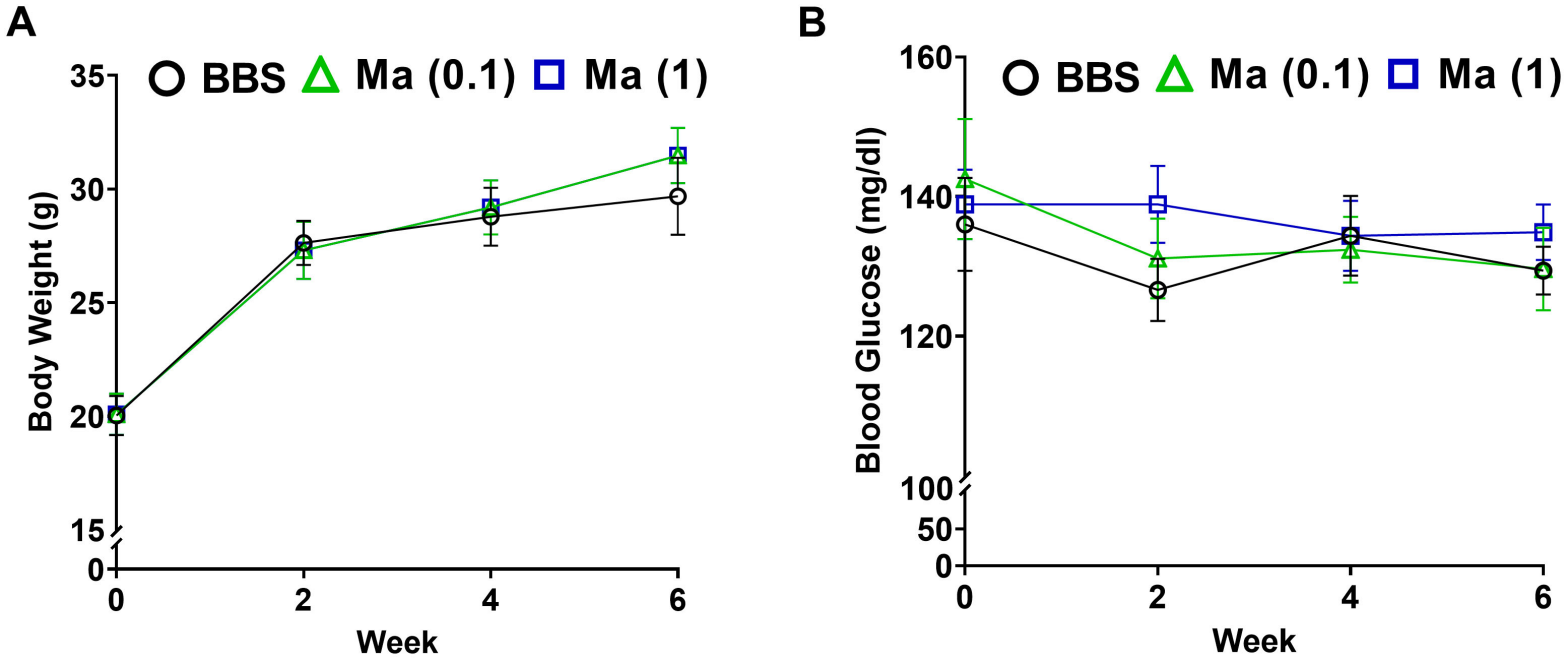
Effect HK *M. aurum* treatment on body weight and blood glucose levels in non-diabetic mice. Non-diabetic BALB/c mice were treated with three doses of borate-buffered saline (BBS) or HK *M. aurum* (Ma; 0.1 mg or 1 mg per injection) given 2 weeks apart. Mice **(A)** body weights and **(B)** blood glucose levels were recorded on a weekly basis for different mouse groups. Each symbol represents the mean value ± SEM of body weight or blood glucose level for each mouse group (n = 8 mice per group). Statistically significant differences were determined by two-way ANOVA followed by Tukey *post-hoc* test.

The primary set of experiments was carried out to evaluate the potential effect of HK *M. aurum* administration to non-diabetic BALB/c mice on their body weight and blood glucose levels. Non-diabetic mice were injected with three doses of 0.1 or 1 mg per injection of HK *M. aurum* given 2 weeks apart. Body weight and blood glucose levels were monitored on a weekly basis up to week 6 and compared to control non-diabetic mice that were injected with BBS as vehicle. Both parameters were found to be similar between the two mouse groups, whereby neither body weights (**Figure 1A**) nor blood glucose levels (**Figure 1B**) were significantly altered in non-diabetic mice treated with 0.1 mg or 1 mg per injection of HK *M. aurum* mice.

**Figure 2:**
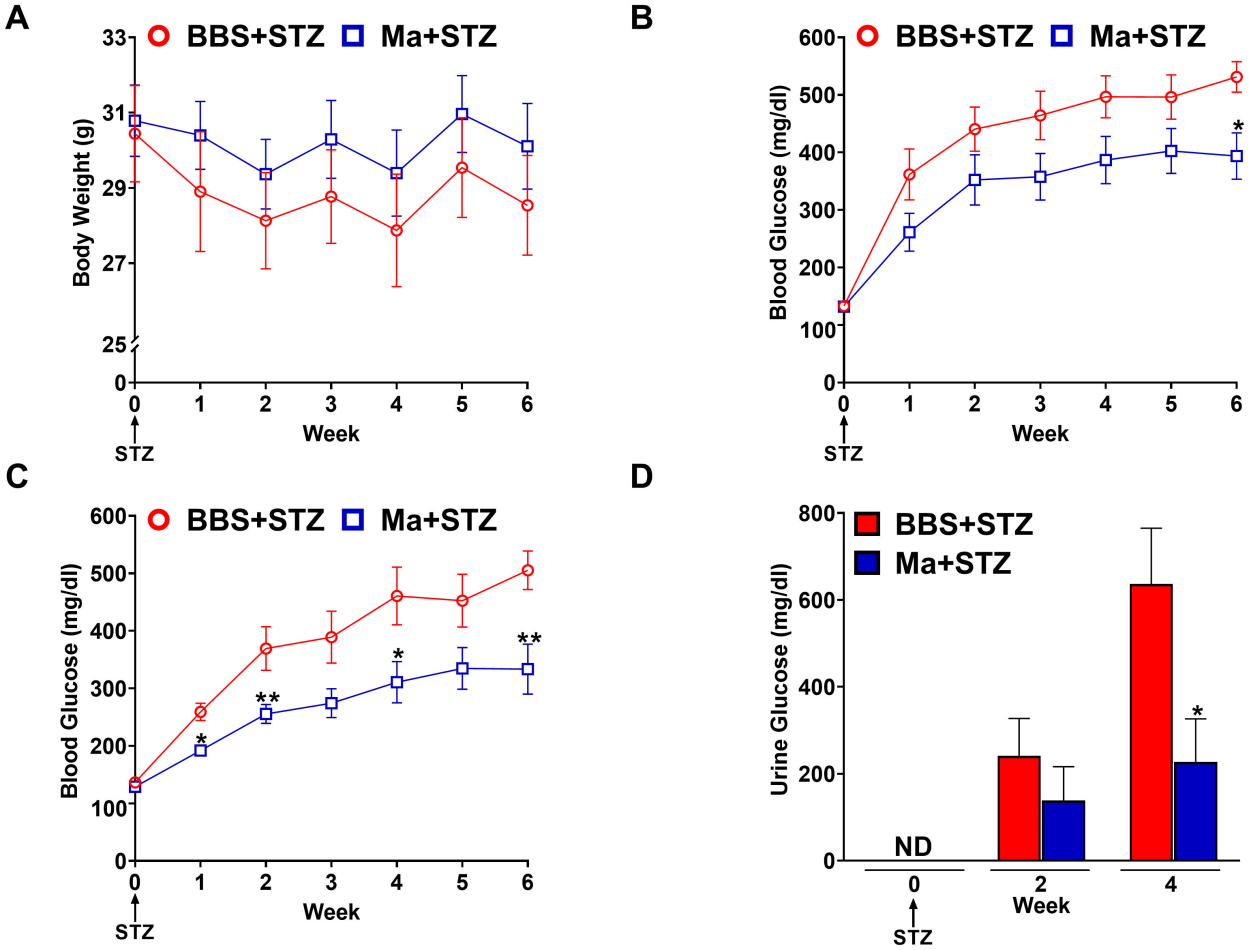
Prophylactic effects HK *M. aurum* on body weight, blood, and urine glucose levels in STZ-induced diabetic mice. Non-diabetic BALB/c mice were treated with three doses of borate-buffered saline (BBS) or HK *M. aurum* (Ma; 1 mg per injection) given 2 weeks apart. After 6 weeks of prophylactic treatment (at week 0), diabetes was induced in both groups of mice through injecting them with STZ (150 mg/kg). The control non-diabetic group received citrate buffer (BBS + CB). Mice **(A)** body weights and **(B, C)** blood glucose levels were measured on a weekly basis up to 6 weeks after STZ, whereas their **(D)** urine glucose levels were analyzed only at weeks 2 and 4 after STZ. **(A–C)** Each symbol represents the mean value ± SEM of body weight or blood glucose level for each mouse group (A, B: n = 12 mice per group; C: n = 7 mice per group). **(D)** Bar graphs represent the mean value ± SEM of urine glucose levels for each mouse group (n = 12 mice per group). For body weights and blood glucose levels, statistically significant differences were determined by two-way ANOVA followed by Tukey *post-hoc* test. For urine glucose levels, statistically significant differences were determined by multiple unpaired t-tests. *p < 0.05 and **p < 0.01 *versus* the BBS + STZ group. ND, not detected.

Next, we investigated the potential prophylactic effect of HK *M. aurum* in restoring weight gain and reducing hyperglycemia in STZ-induced diabetic mice. Mice were pre-treated with three doses of 1 mg per injection of HK *M. aurum* given 2 weeks apart prior to STZ injection. STZ was administered 6 weeks after the first *M. aurum* dose. As expected, the diabetic group (BBS + STZ) was unable to gain weight on a weekly basis as it did prior to STZ injection and HK *M. aurum* did not seem to prevent the STZ-induced halt in body weight gain (**Figure 2A**). Blood glucose levels were monitored on a weekly basis in *M. aurum*–pre-treated group (Ma + STZ) and compared to those of control diabetic group (BBS + STZ). The HK *M. aurum*–pre-treated group showed a trend toward decreased blood glucose levels for 5 weeks after STZ injection, whereas, at week 6, the HK *M. aurum*–pre-treated group had a significantly (*p* < 0.05) lower (~26%) blood glucose level than its corresponding diabetic control mice group (**Figure 2B**). Further analysis of mice subpopulations in both groups revealed that HK *M. aurum* pre-treatment exerted a more evident blood glucose–lowering effect in terms of statistical significance (*p* < 0.05) at weeks 1, 2, 4, and 6 when severely diabetic mice (blood glucose > 300 mg/dL) (24) within the first week after STZ injection were excluded from analysis. Moreover, a statistically significant (*p* < 0.01) decrease of ~34% in glycemic levels was observed at week 6 after STZ injection in mice pre-treated with HK *M. aurum* (1 mg per injection) as compared to that in the BBS pre-treated group (**Figure 2C**). In confirmation to the results observed on blood glucose levels, urine glucose levels were monitored for 4 weeks after STZ injection. The *M. aurum*–pre-treated diabetic group (Ma + STZ) showed a trend toward a decrease in urine glucose levels at week 2 that became statistically significant (*p* < 0.05) at week 4 after STZ injection with urine glucose levels that were 2.8-fold lower than those of the diabetic group pre-treated with BBS (BBS + STZ) (**Figure 2D**). We also noted that pre-treating mice with a lower dose of HK *M. aurum* (0.1 mg per injection) failed to restore weight gain and to reduce hyperglycemia in STZ-induced diabetic mice (**Supplementary Figure 2**).

### Prophylactic treatment with HK *M. aurum* prevents the dysregulation of skeletal muscle UCP3 and liver LDH and UCP2 but fails to restore liver GLUT2 and skeletal muscle GLUT4 protein expression levels in STZ-induced diabetic mice

**Figure 3:**
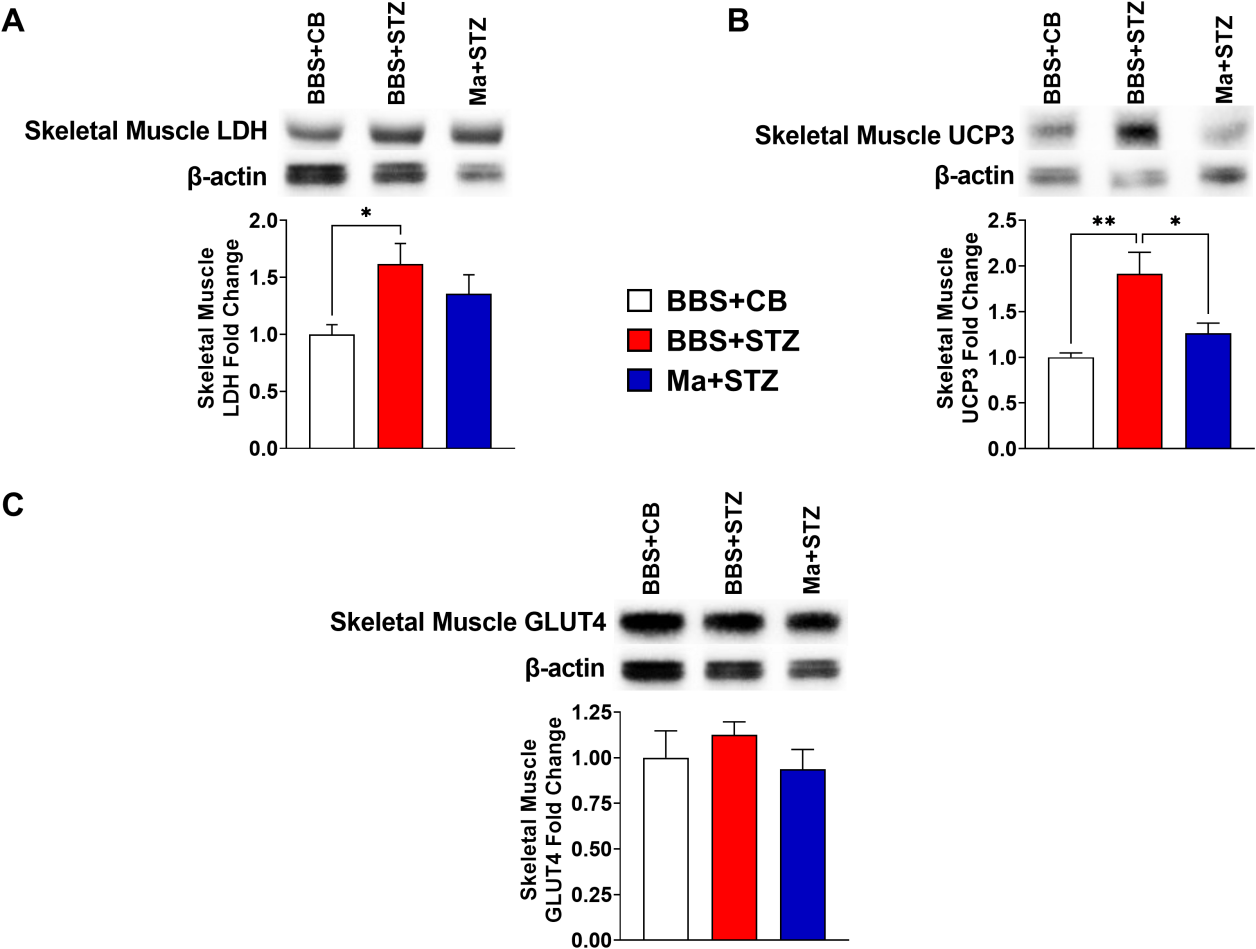
HK *M. aurum* prophylactic treatment prevents the upregulation of skeletal muscle UCP3 expression in STZ-induced diabetic mice. Non-diabetic BALB/c mice were treated with three doses of borate-buffered saline (BBS) or HK *M. aurum* (Ma; 1 mg per injection) given 2 weeks apart. After 6 weeks of prophylactic treatment (at week 0), diabetes was induced in both groups of mice through injecting them with STZ (150 mg/kg). The control non-diabetic group received citrate buffer (BBS + CB). Representative Western blots showing protein expression levels and quantification of skeletal muscle **(A)** LDH, **(B)** UCP3, and **(C)** GLUT4 protein expression levels were performed in normal non-diabetic (BBS + CB), untreated diabetic (BBS + STZ), and Ma-treated diabetic (Ma + STZ) mice at week 6 after STZ. Bar graphs show the relative protein density of **(A)** LDH, **(B)** UCP3, and **(C)** GLUT4 after normalization with β-actin protein. Data are expressed as mean ± SEM (n = 5–10 mice per group). One-way ANOVA test followed by the Tukey’s multiple comparison *post-hoc* test was used to compare the means between the three different experimental groups. Differences between groups were considered to be statistically significant at *p < 0.05 and **p < 0.01.

**Figure 4:**
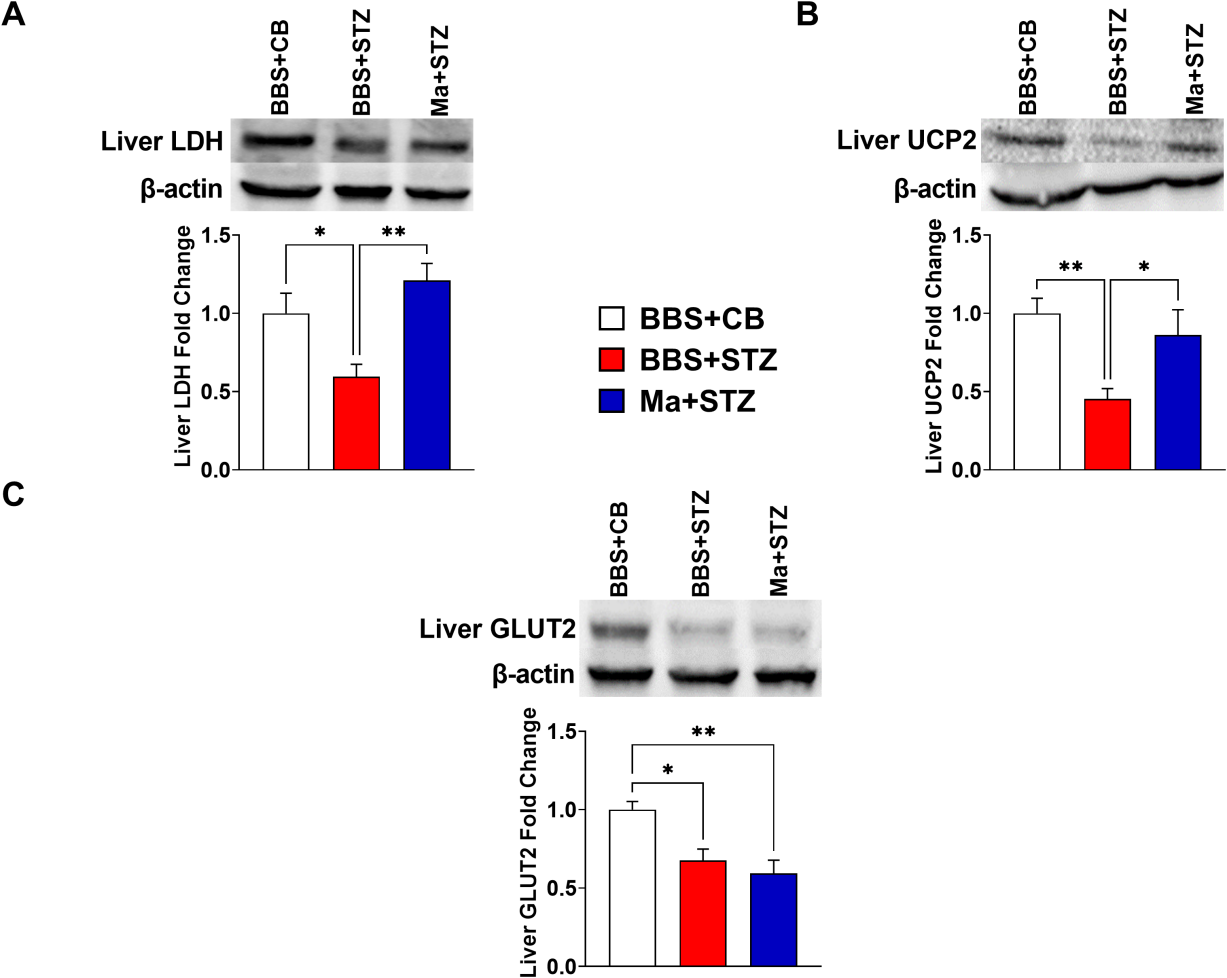
HK *M. aurum* prophylactic treatment prevents the downregulation of liver LDH and UCP2 expression in STZ-induced diabetic mice. Non-diabetic BALB/c mice were treated with three doses of borate-buffered saline (BBS) or HK *M. aurum* (Ma; 1 mg per injection) given 2 weeks apart. After 6 weeks of prophylactic treatment (at week 0), diabetes was induced in both groups of mice through injecting them with STZ (150 mg/kg). The control non-diabetic group received citrate buffer (BBS + CB). Representative Western blots showing protein expression levels and quantification of liver **(A)** LDH, **(B)** UCP2, and **(C)** GLUT2 in normal non-diabetic (BBS + CB), untreated diabetic (BBS + STZ), and Ma-treated diabetic (Ma + STZ) mice at week 6 after STZ. Bar graphs show the relative protein density of **(A)** LDH, **(B)** UCP2, and **(C)** GLUT2 after normalization with β-actin protein. Data are expressed as mean ± SEM (n = 5–11 mice per group). One-way ANOVA test followed by the Tukey’s multiple comparison *post-hoc* test was used to compare the means between the three different experimental groups. Differences between groups were considered to be statistically significant at *p < 0.05 and **p < 0.01.

Western blot was performed to assess the effect of HK *M. aurum* prophylactic treatment on the expression of key metabolism–related proteins that are involved in glucose transport (GLUT2 and GLUT4), glycolysis (LDH), and mitochondrial uncoupling (UCP2 and UCP3). Therefore, the expression levels of the aforementioned proteins were evaluated in the skeletal muscle and/or liver of normal control mice (BBS + CB), untreated diabetic mice (BBS + STZ), and *M. aurum*–treated diabetic mice (Ma + STZ). In the skeletal muscle, LDH and UCP3 protein levels were shown to be significantly elevated in the untreated diabetic group (BBS + STZ) as compared to that in the non-diabetic control group (BBS + CB) (**Figures 3A, B**). Prophylactic treatment with HK *M. aurum* significantly reduced the STZ-induced elevation in UCP3 protein expression in the Ma + STZ group (**Figure 3B**); however, such treatment did not affect LDH protein expression (**Figure 3A**). In comparison to control non-diabetic mice (BBS + CB), the protein expression of skeletal muscle GLUT4 was not significantly affected by STZ treatment in the diabetic group (BBS + STZ) as well in the HK *M. aurum–*pre-treated diabetic group (Ma + STZ) (**Figure 3C**). Results showed that hepatic LDH and UCP2 protein expression levels were significantly (*p* < 0.05) downregulated in the untreated diabetic group (BBS + STZ) as compared to that in the control non-diabetic group (BBS + CB) (**Figures 4A, B**). Prophylactic treatment of diabetic mice with HK *M. aurum* (BBS + STZ) prevented the STZ-induced downregulation in LDH and UCP2 levels, whereby the expression levels of both proteins were comparable to those of control non-diabetic mice (BBS + CB) but significantly (*p* < 0.05) higher than those of untreated diabetic mice (STZ + BBS) (**Figures 4A, B**). GLUT2 expression in the liver was found to be reduced by ~33% in the untreated diabetic group (BBS + STZ) as compared to the control non-diabetic group (BBS + CB) (**Figure 4C**). However, HK *M. aurum* pre-administration to diabetic mice (Ma + STZ) did not affect GLUT2 protein expression as compared to the untreated diabetic mice (BBS + STZ) (**Figure 4C**).

## Discussion

Our study explored the potential prophylactic anti-diabetic effects of multiple doses of HK *M. aurum* in STZ-induced diabetic mice. HK *M. aurum* was administered to mice 6 weeks prior to diabetes induction by a single high-dose STZ treatment, which induces T1D. This approach was designed to evaluate whether HK *M. aurum* could prevent or ameliorate hyperglycemia in STZ-induced diabetic mice. We have demonstrated for the first time that prophylactic treatment with three doses of HK *M. aurum* is able to significantly reduce hyperglycemia and glycosuria in STZ-induced diabetic mice. Meanwhile, prophylactic treatment with HK *M. aurum* corrected the dysregulated protein levels of liver LDH and UCP2 and skeletal muscle UCP3 in STZ-induced diabetic mice. To model diabetes in BALB/c mice, we utilized STZ, a compound widely used to induce diabetes in rodents by selectively targeting pancreatic β-cells (25). STZ is a glucosamine-nitrosourea compound that enters pancreatic β-cells via the GLUT2 glucose transporter. Once inside the cells, STZ causes β-cell death through multiple mechanisms that involve the induction of DNA damage and oxidative stress, thus contributing to the apoptosis and necrosis of pancreatic β-cells (26). These mechanisms collectively lead to a severe reduction in insulin secretion and the development of subsequent hyperglycemia and therefore mimicking aspects of T1D when a single high dose (150 mg/kg) of STZ is injected into BALB/c mice.

The complex configuration of the cell wall of various mycobacterial species has granted them potent immunomodulatory activities (4). The live attenuated strain of *M. bovis*, BCG, has been reported to hold immunomodulatory effects that target both the innate and adaptive arms of the human immune system (27). In that manner, BCG is able to reduce hyperglycemia in patients with diabetes and in STZ-induced diabetic mice by inducing Treg and decreasing Tc cell numbers. Furthermore, it is able to reprogram Tregs’ metabolism to favor glucose take-up and oxidation through glycolysis (12, 13). Another mycobacterial species, *M. vaccae*, that was used in the form of a HK whole-cell preparation, has gained attention as an effective immunomodulator with evident effects on adaptive immune cells, via Treg expansion (18), as well as on innate immune cells (19, 28). Currently, a HK preparation of *M. aurum* is being marketed as a promising “tuner” of the immune system, whereby it might aid in coping with anxiety, managing undesired inflammatory responses, and enhancing stress resilience (21). Whole-genome sequencing coupled with phylogenetic analysis revealed a high degree of similarity between *M. vaccae* and *M. aurum*; thus, it is highly likely that the latter species might possess comparable immunomodulatory properties and probably a potential benefit in the management of diabetes. Therefore, lessons learned from studies highlighting the prophylactic anti-diabetic effects of BCG have helped us to construct our study design in the hope to uncover similar properties of HK *M. aurum* in preventing or lowering hyperglycemia in STZ-induced diabetic mice.

In the present study, non-diabetic mice prophylactically treated with three intradermal doses of HK *M. aurum* did not display any significant changes in their glycemic levels or weight gain patterns, thus strengthening the safety profile of this HK mycobacterial preparation. This is in accordance with a previous toxicity evaluation study in rats that confirmed that HK *M. aurum* was safe and tolerable when administered via the oral route, hence qualifying it as a possible food ingredient (22).

As expected, data from the current study revealed that untreated STZ-induced diabetic mice displayed an increase in their blood glucose levels (> 300 mg/dL) starting week 1 after STZ injection as well as a retardation in their body-weight gain. However, prophylactic treatment of diabetic mice with three doses of HK *M. aurum* (1 mg per injection) resulted in a trend toward a decrease in blood glucose levels as of week 1 until week 5 after STZ injection with a significant reduction in urine and blood glucose levels at week 4 and week 6, respectively, after STZ injection. A similar hyperglycemia-lowering effect has been previously reported with BCG, whereby a single footpad prophylactic administration of BCG to STZ-induced diabetic BALB/c mice led to a significant decline in their blood glucose levels as compared to those in untreated STZ-induced diabetic mice (12).

Further examination of subpopulations of different mouse groups in this study resulted in an intriguing finding, whereby when severely hyperglycemic mice (blood glucose levels ≥ 300 mg/dL) (24) were excluded from data analysis at week 1 after STZ injection, the blood glucose–lowering effect of HK *M. aurum* became more evident and statistically significant (*p* < 0.05). In this case, diabetic mice prophylactically treated with HK *M. aurum* had blood glucose levels that were constantly lower by an average of ~30% throughout the 6 weeks after STZ injection, as compared to untreated diabetic mice. This observation might be attributed to the fact that HK *M. aurum* pre-treatment would be less effective in delaying or reducing severe hyperglycemia in mice exhibiting a sharp and rapid rise in their blood glucose levels.

HK *M. aurum* was more effective at lowering hyperglycemia when administered at higher doses (1 mg per injection *versus* 0.1 mg per injection). Lower doses might not be able to generate an immune response capable of inducing systemic modifications to the host’s metabolic status and therefore improving glycemic control. Dose-dependent effects have been also reported in studies that employed BCG to prevent diabetes in non-obese diabetic (NOD) mouse (8). This proposed process might involve but is not restricted to the induction of TNF-α, an essential cytokine that indicates that an active and sufficient mycobacterial-induced immune response is being mounted (10, 17, 29, 30). We opted to use multiple doses of HK *M. aurum* so as to maximize the possible desirable effects. Multiple therapeutic doses of BCG have been shown to be more effective in human diabetes studies, as they probably increase the likelihood of “resetting” the host’s immune system (13, 31). In addition, a lag time between prophylactic treatment with BCG and STZ-induced hyperglycemia has been found to be crucial for BCG to exert its hyperglycemia-lowering effect in STZ-induced diabetic mice (12, 32). Similarly, BCG therapy has been shown to improve HbA1c levels and to decrease insulin usage in patients with T1D following a lag period of 3 years after BCG injection (12). Given that our study is the first to use HK *M. aurum* in the context of preventing or ameliorating hyperglycemia, we factored in the possibility of a similar lag time being required for its efficacy. Our study revealed the benefit of administering three doses of HK *M. aurum* 6 weeks prior to STZ injection.

Although HK *M. aurum* treatment resulted in lower blood glucose levels in the treated diabetic group as compared to those in the untreated diabetic group, blood glucose levels still remained ≥ 200 mg/dL. This observation indicates that, although HK *M. aurum* appears to mitigate the severity of hyperglycemia, it does not completely normalize blood glucose levels. The observed partial hyperglycemia-lowering efficacy suggests that HK *M. aurum* may help to reduce the extent of glucose elevation but may not be sufficient to fully restore normal glucose homeostasis under the given treatment conditions. This warrants further experiments that investigate the dosing, timing and alternative routes of administration so as to gain a better understanding of HK *M. aurum*’s beneficial properties in diabetes prevention or management.

Insulin sensitive tissues such as the liver and skeletal muscle are naturally the most affected from insulin insufficiency and hyperglycemia (33). In the liver, LDH and GLUT2 are the two important proteins involved in glucose metabolism and regulation. When blood glucose levels are high, LDH levels in the liver can increase to support the higher demand for glucose oxidation. However, prolonged hyperglycemia can lead to oxidative stress and damage to liver cells, which can further result in decreased LDH levels and impaired liver function. Furthermore, insulin insufficiency in the liver leads to a decrease in glucokinase and fructose 2,6-bisphosphate that inhibits glycolysis while increasing enzymes related to glycogen breakdown and gluconeogenesis such as glucose-6-phosphatase (34–36).

GLUT2 is a high-capacity, low-affinity glucose transporter predominantly expressed in the liver, pancreas, intestine, and kidneys (37). It plays a crucial role in the regulation of glucose homeostasis. In hepatocytes, GLUT2 facilitates the bidirectional transport of glucose, allowing the liver to uptake glucose during hyperglycemia and release glucose during hypoglycemia (38). Its activity is primarily regulated by the concentration gradient of glucose rather than insulin levels. Although GLUT2 is considered insulin-independent, its expression and function can be modulated by the metabolic state of the organism. For instance, hyperglycemia can lead to an increase in GLUT2 expression as part of the body’s adaptive mechanism to manage elevated blood glucose levels (39). Given that STZ-induced diabetes leads to significant hyperglycemia, assessing GLUT2 expression helps us understand how HK *M. aurum* prophylactic treatment might influence hepatic glucose handling under diabetic conditions.

In our study, liver GLUT2 and LDH levels were found to be reduced in untreated diabetic mice 6 weeks after STZ injection. GLUT2 provides a major route for excess blood glucose to access the liver; thus, any reduction in its expression would contribute to further elevation of hyperglycemia (36, 37). Moreover, any decrease in the glycolytic activity in the liver, as evidenced by a reduction in LDH levels, would eventually decrease glucose usage and promote hepatic glucose production and output that is further translated by the release of glucose into the blood stream despite the elevated blood glucose levels. HK *M. aurum* prophylactic treatment of diabetic mouse group prevented the downregulation in liver LDH levels. This is indicative of increased glucose utilization by the liver and would probably reflect an improved liver function. However, the noted increase in LDH levels was not accompanied with a correction (upregulation) of liver GLUT2 levels in the *M. aurum*–pre-treated diabetic group. In other words, the decrease in hepatic glucose production, which leads to a reduction in glucose export out of the liver, possibly explains why the *M. aurum*–pre-treated diabetic group had lower blood glucose levels than the untreated diabetic group. In addition, *M. aurum* might exert its hyperglycemia-lowering effect on liver glucose production independently of changes in GLUT2-dependent glucose import/export.

UCP2 is a mitochondrial transporter protein that is able to dissipate the proton gradient across the inner mitochondrial membrane in the presence of certain activators such as fatty acids, coenzyme Q, superoxide, and lipid peroxidation products (40). This process uncouples oxidative phosphorylation from ATP production (40). UCP2 is also known to act as a regulator of mitochondrial reactive oxygen species (mitoROS) by means of a negative feedback loop (41–43). Stimulation of murine macrophages by the mycobacterial cell wall component, muramyl dipeptide, rapidly upregulates the expression of UCP2 and pre-treatment with vitamin E attenuates the upregulation of UCP2 (44). An increase in mitoROS upregulates UCP2 expression, which, in turn, dissipates the membrane potential, making the electron transport chain (ETC) less reduced and therefore less prone to produce free radicals. Under conditions of prolonged hyperglycemia, UCP2 expression in the liver can be downregulated (45). Chronic hyperglycemia can lead to the production of ROS, which can impair mitochondrial function by oxidative stress and consequently results in a decrease in liver enzymes and function. In this study, liver UCP2 expression was found to be downregulated in control diabetic mice. Nevertheless, prophylactic treatment of diabetic mice with HK *M. aurum* protected the liver from the disruptive effects of prolonged hyperglycemia. Just like liver LDH, the expression of UCP2 in the treated diabetic group was comparable to the normoglycemic group. Being a negative regulator of ROS, an increase in UCP2 levels in the HK *M. aurum*–pre-treated diabetic group indicates that the mitochondrial anti-oxidative mechanisms in the liver are maintained despite chronic hyperglycemic conditions.

In the skeletal muscle, UCP3, another member of the uncoupling protein family, and LDH levels were markedly increased in the control diabetic group as compared to those in the non-diabetic group. This goes in accordance with similar findings in the literature where upregulated UCP3 and LDH levels have been reported during states of insulin insufficiency, diabetes, and fasting (46, 47). Research on UCP3 suggests its involvement in metabolic regulation, particularly in the utilization of fatty acids as a fuel source. UCP3 may play a role in protection against lipid-induced oxidative stress and in regulation insulin sensitivity (48). On one hand, insulin insufficiency was found to cause an upregulation in key enzymes involved in glycolysis and glycogenolysis, justifying the observed increase in LDH levels in the skeletal muscle of diabetic mice (33, 49–51). On the other hand, free fatty acid (FFA) uptake is increased along with a partially inactive β-oxidation pathway, thus leading to the FFA accumulation near the mitochondria and subsequently triggering ROS-induced peroxidation and elevation in the levels of lipid peroxide, a known positive activator of UCP3 (52). Therefore, an increase in UCP3 levels serves as a type of feedback inhibition to export excess FFAs and to reduce ROS levels through UCP3-mediated uncoupling (53–55).

In our study, we observed that LDH expression in the skeletal muscle was elevated in the untreated diabetic group, whereas it was decreased in the liver. Elevated LDH expression in the skeletal muscle could be attributed to increased anaerobic glycolysis, which serves as a compensatory mechanism for energy production in the absence of adequate insulin. This suggests that skeletal muscle tissue adapts to the insulin-deficient state by enhancing anaerobic pathways to meet its energy demands. Conversely, the decrease in LDH expression observed in the liver may reflect altered metabolic functions in hepatic tissue under diabetic conditions. This could be due to compromised liver function or shifts in metabolic pathways that reduce the reliance on glycolysis. The liver, being central to various metabolic processes, might exhibit a different adaptive response to diabetes compared to skeletal muscle, potentially prioritizing gluconeogenesis or other metabolic pathways over glycolysis. Our results showed that HK *M. aurum* prophylactic treatment can prevent the STZ-induced upregulation in UCP3 expression with no effect on LDH expression. The observed *M. aurum*–induced reduction in the skeletal muscle UCP3 expression in diabetic mice might be also attributed to a diminished level of ROS-induced peroxidation or translocation of FFAs to the mitochondria.

Another GLUT member, GLUT4, is predominantly found in the skeletal muscle and adipose tissue, where it mediates insulin-stimulated glucose uptake (56). Under basal conditions, GLUT4 resides in intracellular vesicles and translocates to the plasma membrane in response to insulin signaling (57). Evaluating GLUT4 in basal conditions (i.e., without acute insulin stimulation) provides insights into the chronic regulation of GLUT4 expression and its potential contribution to overall glucose homeostasis during diabetic states. In STZ-induced diabetic mice, insulin levels are significantly reduced due to β-cell destruction, which impacts GLUT4 translocation and glucose uptake. By measuring GLUT4 expression, we aimed to determine whether HK *M. aurum* treatment could modulate GLUT4 levels and potentially enhance glucose uptake in the skeletal muscle despite the low insulin environment. Our results showed that the protein expression of skeletal muscle GLUT4 was not regulated either in untreated or in *M. aurum*–pre-treated diabetic mice as compared to that in non-diabetic mice.

It is important to note that STZ-induced diabetes can involve insulitis, an inflammatory response in the pancreatic islets (58). This response, although different from the autoimmune attack observed in T1D, can contribute to β-cell dysfunction and death. Although our current results do not directly demonstrate an anti-inflammatory effect of HK *M. aurum*, it is plausible that the observed reduction in hyperglycemia could be partially attributed to an anti-inflammatory effect induced by HK *M. aurum*. In fact, *M. vaccae*, which is phylogenetically very close to *M. aurum*, has been reported to possess potent anti-inflammatory properties when used as a HK preparation (59, 60). In these studies, HK *M. vaccae* was shown to induce Tregs and anti-inflammatory cytokine production. Therefore, the possible modulation of inflammation by HK *M. aurum* remains a hypothesis that requires further investigation.

**Figure 5:**
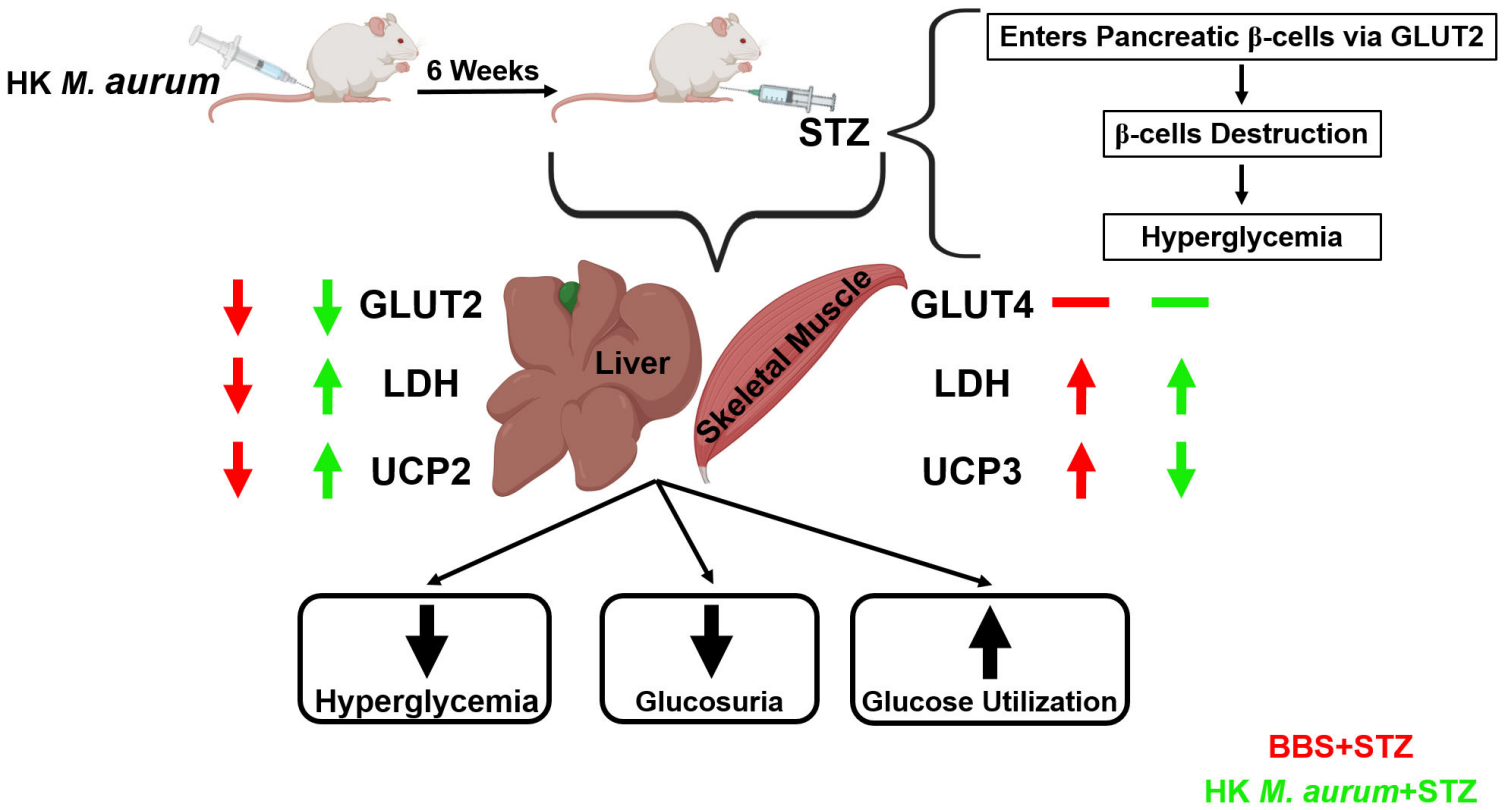
Schematic diagram summarizing the prophylactic anti-diabetic effects of HK *M. aurum* in STZ-induced diabetic mice.

In summary, HK *M. aurum* exerted a hyperglycemia-reducing effect in STZ-induced diabetic mice via a selective mechanism that was only evident under hyperglycemic conditions. This highlights HK *M. aurum* safety and its potential use for the amelioration of hyperglycemia and diabetes. Prophylactic treatment with HK *M. aurum* was shown to increase glucose utilization in hyperglycemic conditions independently from the impact of glucose import to the liver via GLUT2 and as evidenced by LDH expression. It was also able to regulate the expression of UCP2 and UCP3 in the liver and skeletal muscle, respectively, of STZ-induced diabetic mice. This might reflect an improvement in liver and skeletal muscle metabolic function and anti-oxidative mechanisms (**Figure 5**). These findings underscore a potential prophylactic value of HK *M. aurum* in managing diabetes development and warrant additional investigation into its underlying mechanisms of action for later clinical development. Future research should be conducted to further evaluate the therapeutic effects of HK *M. aurum* in STZ-induced diabetic mice.

## Data Availability

The original contributions presented in the study are included in the article/**Supplementary Material**. Further inquiries can be directed to the corresponding author.
