# Supplementary material for: Reduction of hyperglycemia in STZ-induced diabetic mice by prophylactic treatment with heat-killed Mycobacterium aurum: possible effects on glucose utilization, mitochondrial uncoupling, and oxidative stress in liver and skeletal muscle
Source: Front Endocrinol (Lausanne). 2024 Sep 6;15:1427058. doi: 10.3389/fendo.2024.1427058 (PMC11456689; doi:10.3389/fendo.2024.1427058)
Supplement: Supplementary file 1 [file DataSheet1.pdf]

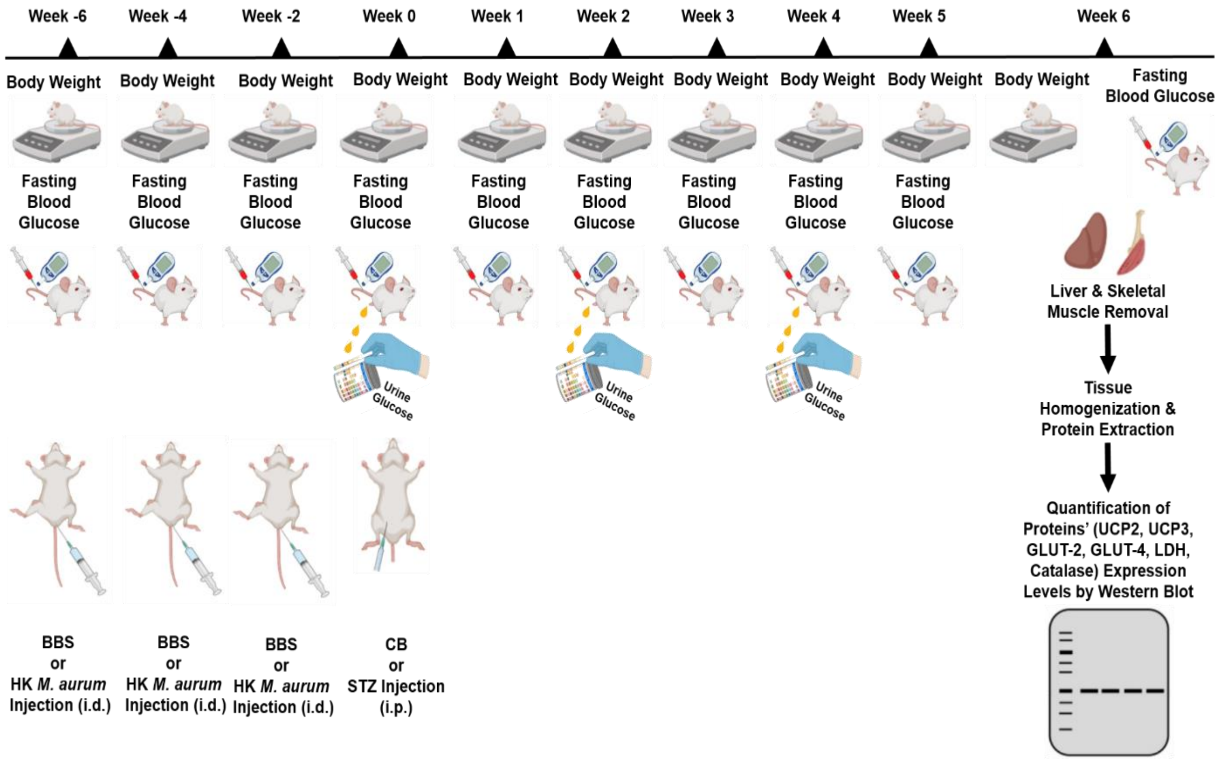

Supplementary Figure 1. Mouse experimental procedures

s

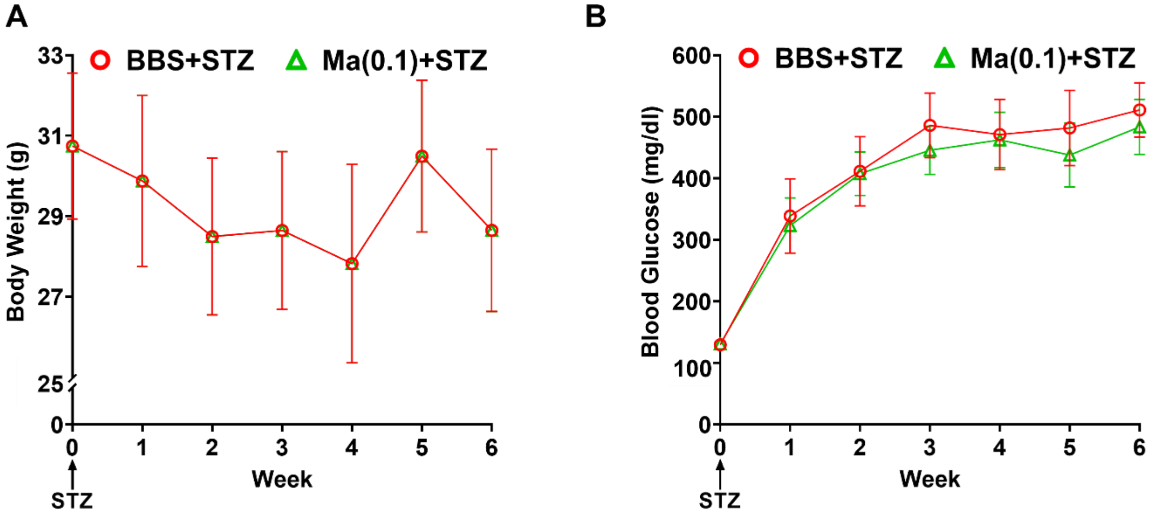

Supplementary Figure 2. Prophylactic effects HK *M. aurum* (0.1 mg/injection) on body weight and blood glucose levels in STZ-induced diabetic mice. Non-diabetic BALB/c mice were treated with 3 doses of borate buffered saline (BBS) or HK *M. aurum* (Ma; 0.1 mg/injection) given 2 weeks apart. After 6 weeks of prophylactic treatment (at week 0), diabetes was induced in both groups of mice through injecting them with 150 mg/kg of STZ. Control non-diabetic group received citrate buffer (BBS+CB). Mice (A) body weights and (B) blood glucose levels were measured on weekly basis up to 6 weeks post-STZ. Each symbol denotes the mean value ± SEM of body weight or blood glucose level for each mouse group (n=7 mice/group).
